# Metabolic drift in the aging brain

**DOI:** 10.18632/aging.100961

**Published:** 2016-05-15

**Authors:** Julijana Ivanisevic, Kelly L. Stauch, Michael Petrascheck, H. Paul Benton, Adrian A. Epstein, Mingliang Fang, Santhi Gorantla, Minerva Tran, Linh Hoang, Michael E. Kurczy, Michael D. Boska, Howard E. Gendelman, Howard S. Fox, Gary Siuzdak

**Affiliations:** ^1^ Metabolomics Research Platform, University of Lausanne, 1005 Lausanne, Switzerland; ^2^ Departments of Pharmacology and Experimental Neuroscience, University of Nebraska Medical Center, Omaha, NE 68198-5800, USA; ^3^ Departments of Chemical Physiology, Molecular and Cellular Neuroscience, The Scripps Research Institute, La Jolla, CA 92037, USA; ^4^ Scripps Center for Metabolomics and Mass Spectrometry, Departments of Chemistry, Molecular and Computational Biology, The Scripps Research Institute, La Jolla, CA 92037, USA; ^5^ Department of Radiology, University of Nebraska Medical Center, Omaha, NE 68198-1045, USA; ^6^ School of Civil and Environmental Engineering, Nanyang Technological University, Singapore 639798, Singapore; ^7^ Drug Metabolism and Pharmacokinetics, Innovative Medicine, AstraZeneca, Mölndal 431 83, Sweden

**Keywords:** metabolic drift, healthy brain aging, metabolomics, energy metabolism, proteomics

## Abstract

Brain function is highly dependent upon controlled energy metabolism whose loss heralds cognitive impairments. This is particularly notable in the aged individuals and in age-related neurodegenerative diseases. However, how metabolic homeostasis is disrupted in the aging brain is still poorly understood. Here we performed global, metabolomic and proteomic analyses across different anatomical regions of mouse brain at different stages of its adult lifespan. Interestingly, while severe proteomic imbalance was absent, global-untargeted metabolomics revealed an energy *metabolic drift* or significant imbalance in core metabolite levels in aged mouse brains. Metabolic imbalance was characterized by compromised cellular energy status (NAD decline, increased AMP/ATP, purine/pyrimidine accumulation) and significantly altered oxidative phosphorylation and nucleotide biosynthesis and degradation. The central energy metabolic drift suggests a failure of the cellular machinery to restore *metabostasis* (metabolite homeostasis) in the aged brain and therefore an inability to respond properly to external stimuli, likely driving the alterations in signaling activity and thus in neuronal function and communication.

## INTRODUCTION

Recent findings from evolutionary genomic studies have stimulated a rethinking of brain energy metabolism and the role it plays in brain function [1]. These findings indicate a significant enrichment in the expression of genes involved in energy metabolism in the human brain when compared to other (nonhuman) primates [2, 3]. The high metabolic cost of the human brain, reaching 20% of the whole body energy consumption, is related to the emergence of higher cognitive functions [4, 5].

Several functional neuroimaging surveys provide evidence that the brain is particularly vulnerable to alterations in energy homeostasis, a trait derived from its oxidative, high-energy demanding metabolism [1, 6, 7]. In vivo monitoring of brain metabolism, using PET (Positron Emission Tomography) and MRS (Magnetic Resonance Spectroscopy), have reported an energy deficit determined by abnormally low glucose metabolism (uptake and oxidation) coupled to mitochondrial dysfunction as one of the main, early indicators of age-related changes toward cognitive impairment in the healthy aging brain and neurodegenerative diseases [8-10].

Glucose is the primary source of energy for brain cells where it is almost fully oxidized via oxidative phosphorylation in the mitochondria to produce ATP [11]. Although glucose is a key energetic substrate for the brain, the alternative substrates such as lactate and ketone bodies are used when glucose is in short supply [12, 13]. While neurons require extensive amounts of energy for signaling processes the astrocytes play an essential role in the regulation of brain energy metabolism [1]. Energy metabolism is coordinated through the mitochondria-cytosol link, and the observed cerebral glucose hypometabolism driven by mitochondrial regulatory dysfunction seems to be an early event in the progression of brain aging that hinders the maintenance of cellular homeostasis. Disrupted ion homeostasis, inability to maintain the integrity of cellular compartments, synaptic communication and neuro-transmission directly affects neural function [14, 15]. Although the impairment of mitochondrial energy-transducing capacity leading to loss of cellular homeostasis is an evolutionary conserved phenomenon considered a hallmark of aging, it is still largely unknown why and how the homeostasis is disrupted [16, 17]. Brain aging is a particularly challenging problem to assess and it is not clear whether the mechanisms that have been identified in different animal models (including rodents, flies, molluscs and worms) at the organism level also play a role in central nervous system (CNS) aging.

With the ‘omic technology improvements we are getting closer to understanding the causes and effects of aging at the molecular level. Genome- and proteome-wide views of age-associated changes in the epigenome, in gene and protein expression, have been described in the aging brain in animal models and across human populations [18-23]. However, metabolome-wide studies are scarce and mainly focused on biofluid screening (e.g. blood plasma, cerebrospinal fluid) for biomarker discovery associated with neurodegenerative diseases that implicate accelerated disease-induced aging. Molecular phenotyping at the metabolite level during the aging process has been limited to a small subset of known highly abundant metabolites (e.g. glucose, lactate, N-acetyl aspartate (NAA), myo-Inositol, creatine, choline, glutamate and glutamine) that can be assessed *in vivo,* using PET and MRS [8, 24]. Low sensitivity of MRS and PET provide limited information concerning low abundant and labile metabolites. Global tissue metabolomics could markedly upgrade our understanding of the molecular bases of brain aging by direct and unbiased monitoring of tissue activity across a broad range of small molecules, including low abundant and trace metabolites, from the whole-organ level down to the regional, cellular and sub-cellular level [25, 26]. Specific types of cells (e.g. cell culture) and/or fractions enriched in specific organelles (e.g. mitochondria) can be routinely analyzed due to considerable advancements in instrument sensitivity.

Here we examine brain energy metabolism in order to characterize the role it plays in central nervous system function during the healthy aging process. In mice, as in humans, aged individuals have shown a variety of cognitive and behavioral changes, including deficits in learning and memory [27, 28]. While most studies have addressed changes in energy metabolism of the aging brain in pathological conditions, in the current study we have applied cutting-edge, mass spectrometry-based ‘omic technologies to reveal metabolic changes that are taking place during the normal brain aging. The proteome and metabolome wide profiling of mouse brain at different stages of the life cycle (12, 18 and 24 months) and across different anatomical regions provided insight into a new phenomenon we define as *metabolic drift* in the aging brain. The intrinsic changes in cellular activity of a healthy aging brain were mainly defined by altered oxidative phosphorylation and nucleotide biosynthesis and degradation, with some parallels to metabolic reprogramming in cancer. Characterization of the aging brain phenotype at the metabolite level is an essential step toward understanding how *metabostasis* is changing and thus deducing the mechanisms to limit the effects of aging.

## RESULTS

### Quenching brain energy metabolism

Prior to global metabolomic and proteomic analyses, and to allow for sensitive, brain energy metabolism investigation, focused beam microwave irradiation (FBMI) was applied to the mice to induce instant euthanasia, simultaneously halting enzymes and quenching the metabolic activity in the brain tissue (see Supplemental Experimental Procedures for detailed explanation). FBMI allowed for the preservation of brain tissue, facilitating brain tissue isolation and dissection. The effectiveness of FBMI has been validated with characterized ^1^H-MRS metabolite relationships (low lactate, high NAA) from postmortem tissue followed by proteomic and metabolomic analyses (Figure S1) [29]. Thus, the brain proteome and metabolome was preserved from degradation and/or transformation during the post-mortem delay.

**Figure 1:**
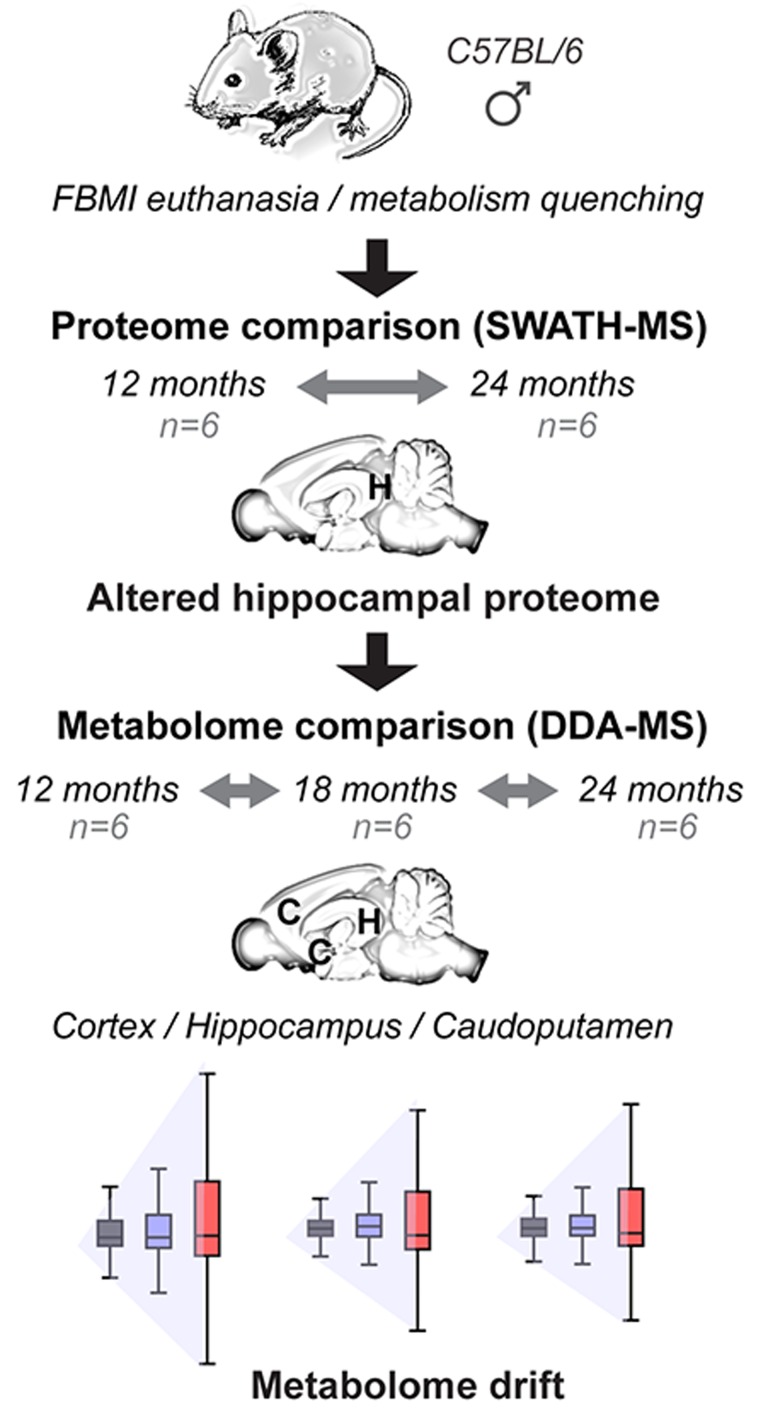
Experimental design of comprehensive regional and temporal profiling of murine brain proteome and metabolome Prior to brain sample collection the Focused Beam Microwave Irradiation (FBMI) was applied as a metabolism quenching technique to inactivate the enzymes and prevent the metabolite degradation during the post-mortem delay (See also Figure S1). Initially the hippocampus was dissected and extracted for proteome profiling using nano SWATH-MS – nano flow data independent tandem mass spectrometry acquisition mode with variable windows to facilitate protein quantification. As the altered hippocampal proteome implied the changes in metabolic processes, the metabolome profiling was performed on three different brain regions using micro HILIC-DDA-MS – capillary flow hydrophilic interaction chromatography coupled with data dependent tandem mass spectrometry acquisition mode to facilitate metabolite identification.

Untargeted proteomic analysis was performed first at two ages, 12 months old (middle aged) and 24 months old (aged) mice. Following the indications from hippocampal proteome analysis the comprehensive metabolomic profiling of central carbon metabolism was performed in the hippocampus and two additional brain regions at these two ages as well as at an intermediate time point, 18 months of age (Figure 1). Water soluble, central carbon metabolites, including energy currency metabolites, were examined by untargeted profiling using hydrophilic interaction chromatography in basic conditions coupled to negative electrospray ionization tandem mass spectrometry (HILIC –ESI-MS/MS).

### Quantitative analysis of the aging hippocampal proteom e implicates metabolic dysfunction

**Figure 2:**
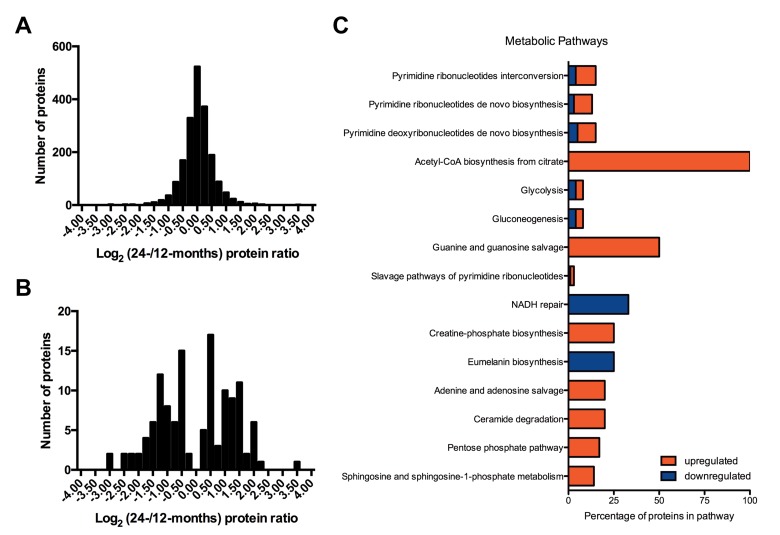
Hippocampal proteomic changes from 12 to 24 months The histogram distribution of the log_2_ (24-/12-month) protein ratios for the (**A**) total 1,925 quantified proteins and (**B**) 126 significantly differentially expressed proteins. (**C**): Bar graph of overrepresented pathways (p < 0.05) based on the protein list of differentially expressed proteins (from 12 to 24 months, 126 proteins from the Bayes-regularized t-test and multiple hypothesis testing corrections). The upregulated and downregulated refer to the percentage of proteins up (increased expression in 24 compared to 12 months) or downregulated (decreased expression in 24 compared to 12 months) in each pathway.

Initially, the proteome wide study of the hippocampus was performed due to its known importance in learning and memory, functions that can decrease with age. SWATH-MS proteomics was used to examine the hippocampal proteome. In total, 1,925 proteins were quantified in all specimens (six independent biological replicates where each hemisphere was analyzed separately) from 12 and 24 month old groups. Overall the majority of the 1,925 proteins were not altered with age in the hippocampus. The distribution of the log_2_ (24-/12-month) protein expression values revealed that 16.4 % of the total proteome experienced a change greater than 1.4 fold (2^±0.5^) with 112 and 204 proteins showing decreased or increased expression with age, respectively (Figure 2A and 2B, Table S1). Of the 1,925 proteins, 6.5% of proteins (126) were determined to be significantly differentially expressed (PPDE ≥ 0.95 and BH ≤ 0.05) between 12- and 24-months of age using a Bayes-regularized t-test and multiple hypothesis testing corrections (Table S2).

The functional assessment of the 126 hippocampal proteins exhibiting altered expression with age using the PANTHER Classification System revealed that the largest group of differentially regulated proteins (24) identified enrichment of the Gene Ontology (GO) biological process term nucleobase-containing compound metabolic process, encompassing cyclic nucleotide, purine, pyrimidine, DNA and RNA metabolic processes (Table S2). Further interrogation of the metabolic pathways representative of these 126 proteins using Ingenuity Pathway Analysis (IPA) revealed that energy currency metabolites and the subunits of biological molecules such as nucleic acids and proteins may be altered with age, thus implying the differences in relative utilization of amino acids, purines, and pyrimidines with aging in the hippocampus (Figure 2C, Figure S2). Overall the proteomic changes in the hippocampus with aging highlighted alterations in mechanisms that have direct impact on the regulation of metabolic processes, particularly nucleobase-containing metabolites.

### Untargeted metabolomics reveals energy metabolic drift in aged mouse brain

**Figure 3:**
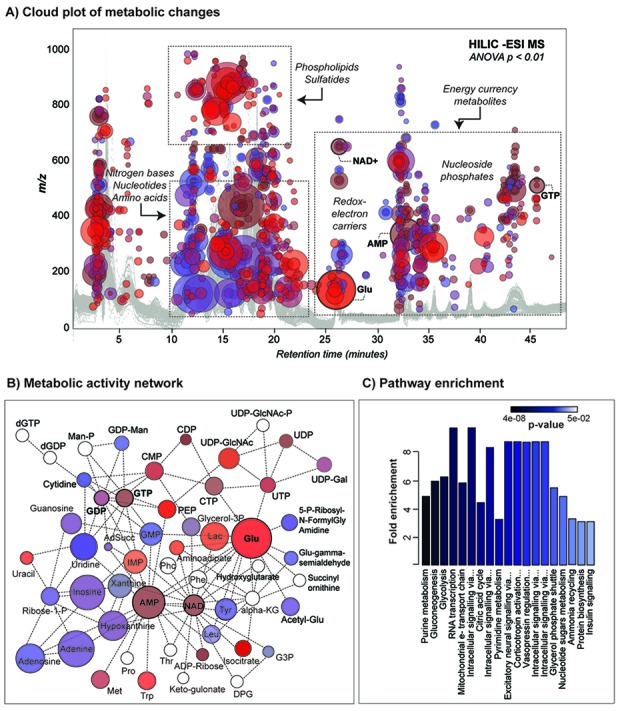
Metabolic changes and associated affected pathways in aged mouse brain using global metabolomic approach (**A**) A cloud plot showing differentially expressed metabolite features (bubbles) across different regions of brain (level of significance: p ≤ 0.01, Intensity > 20,000 ion counts). Metabolite features are projected depending on their m/z ratio and retention time along HILIC gradient. The color of the bubble indicates the level of significance (p-value), with darker color (in blue tones) representing more significant changes (lower p-values). The size of the bubbles is an indicator of ion intensity. A Total Ion Chromatogram (HILIC –ESI MS) is shown in the background. (**B**) Predicted metabolic activity network in healthy aging brain directly from m/z feature table. Network is defined by 4 subnetworks or modules represented by glutamate, AMP, NAD and GDP-GTP that show more internal connections than expected randomly in the whole network. Metabolites are colored according to their p-values and intensities represented above in the cloud plot. Not significantly altered metabolites are represented by transparent bubbles and were included for network connectivity. (**C**) Metabolite set enrichment analysis (MSEA) for mammalian species based on a library containing 88 groups of biologically meaningful metabolite sets. Pathways are ranked depending on the overlap of identified significantly dysregulated metabolites (IDs validated by MS/MS analysis) and total metabolites present in the reference pathway (Table S6).

To characterize the metabolic changes of the healthy aging brain, untargeted metabolomic profiling of hippocampal tissue as well as two additional regions, frontal cortex and caudoputamen was performed at three different stages (12, 18 and 24 months, approximately equivalent to 42, 55 and 69 years in human [46]) of adult mouse lifespan. Using untargeted metabolomics, 818 dysregulated metabolite features (p < 0.01, q < 0.001, Int > 10,000 ion counts) were observed (Figure 3A) when comparing these three different life stages (6 specimens x 3 brain regions at each time point). After removing the chemical and bioinformatic noise, and redundant isotope, adduct, and ion-source fragment features the majority of the putatively identified altered metabolites were associated with the following groups of central core, water soluble metabolites: purines and pyrimidines, amino acids, nucleosides, nucleoside phosphates and redox-electron carriers (see cloud plot in Figure 3A). Among brain lipids, three different sulfatides (from C18 to C24), a major lipid components of myelin sheath, showed a progressive decrease pattern with age (Figure S4). Depletion of myelin-associated glycosphingolipids has been observed to occur upon aging and can be ex-plained by the anatomical changes in neurons including a segmental demyelination. These changes have a negative impact on neuronal plasticity, specifically the information processing and transmission by axons [30].

The disruption of central core metabolism was further explored via network modelling and pathway enrichment analysis. Overall 50 discriminating (*significantly altered*) central core metabolites were identified (using retention time and MS/MS matching as demonstrated in Figure S5) and displayed as readout of the aging brain phenotype (Figure 3B and 3C). The affected metabolic network as a function of age, across all three different brain regions was defined by 4 interacting modules represented by glutamate, AMP, NAD and GDP-GTP. KEGG pathway mapping and metabolite set enrichment analysis for mammalian species highlighted the altered purine and pyrimidine metabolism, and central energy pathways, including glycolysis and oxidative phosphorylation, as well as a few signaling pathways such as adenosine receptor signaling, neural signaling through HTR and serotonin and insulin signaling (Figure 3C).

**Figure 4:**
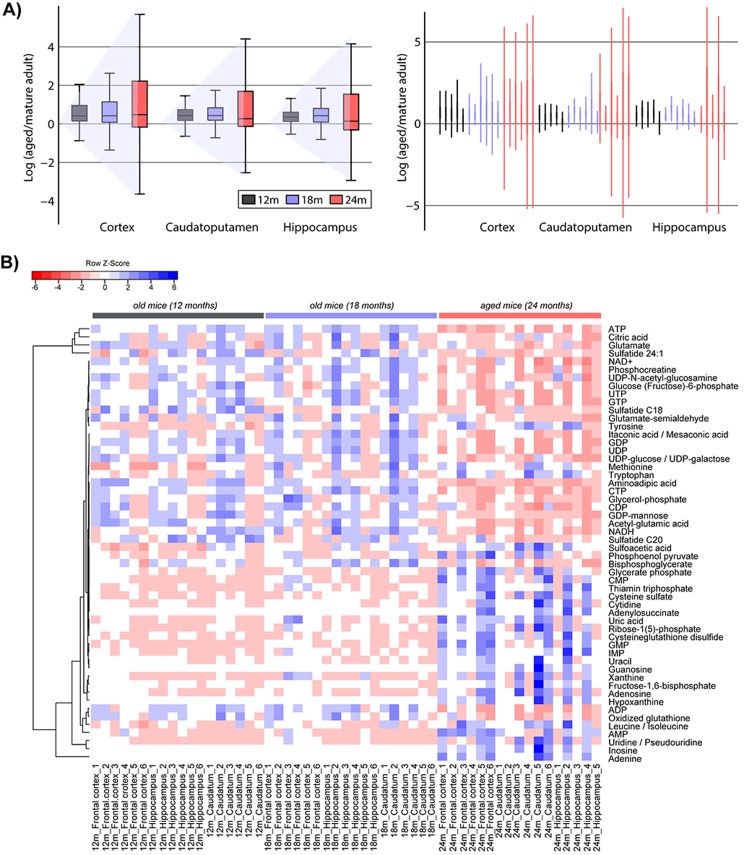
Metabolome drift in the aged murine brain (**A**) Metabolic drift or a significant increase in variance-drift with age, averaged (left panel) and by specimen (right panel) across three different brain regions. The drift was calculated as a log of metabolite fold change from old (24 months) to reference adult stage (12 months). To calculate the drift-variance within the adult reference stage group (12 months) one biological sample in that group was used as reference, the others as experimental samples. This is why we can observe only five individuals at 12 months time point. The only outlier was one hippocampus extract in one 24 months old individual. The increase in metabolome deviation from the adult reference stage (12 months) was evaluated using the level of significance: 12 months *vs*. 18 months for Caudoputamen p = 0.46, for Hippocampus p = 0.02, for Cortex p = 0.06; 18 months *vs*. 24 months p < 10^e-18^ across all brain regions (**B**) Heat map representing the metabolome drift at the metabolite level. Hierarchical clustering was based on the similarity of altered metabolite patterns at three different time points (12, 18 and 24 months, see also Figure S3 for PCA). Discriminating metabolites that represent the readout of the aging process in the brain are shown on the right side (Table S5). Two groups of metabolites whose levels change in opposite directions, either downregulated (upper-right half of the heat map) or upregulated (down-right half of the heat map) in the aged mouse brain can be readily distinguished. MS/MS data matching to facilitate metabolite identification is shown in Figure S5.

Following the recently discovered phenomenon of *transcriptional drift* in several different model organisms (including *C. elegans*, mouse and human) [31], the total dysregulated metabolome data set was further mined. A trend, consistent with transcriptional drift, was revealed in our metabolomic data set and named accordingly as a *metabolic drift* or significant imbalance in metabolite levels in the aged brain when compared to the adult reference stage (Figure 4). Metabolic imbalance in the aged mouse brain was observed in the increased variance of fold changes for the total set of dysregulated metabolites across all three brain regions (hippocampus, cortex, and caudoputamen), without significant difference between regions. (Figure 4A). This increased drift-variance among metabolites within the biological replicates reflects the observed changes of metabolite levels in opposing directions, as shown in the heat map in Figure 4B. Although the deviation away from levels measured in adult reference stage increases with age the metabolic drift in not about the amplitude of variation per metabolite. The metabolic drift has been calculated across the whole pool of affected metabolites, reflecting the variations in opposing directions within the entire metabolite pool and within the same specific pathways. It describes how far the levels of specific metabolites in the aged brain (within the same pathway) have been significantly down-regulated whereas others were significantly up-regulated. The observed changes in opposing directions result in loss of biochemical balance among metabolites within specific functional subsets or biological pathways leading to their disruption and functional decline with age. Age-associated changes in the expression of metabolic enzymes (Figure S2) as well as oxidative modifications that alter enzyme-kinetics also contribute to increasing metabolite imbalance.

### Metabolic drift mapped onto biological pathways

**Figure 5:**
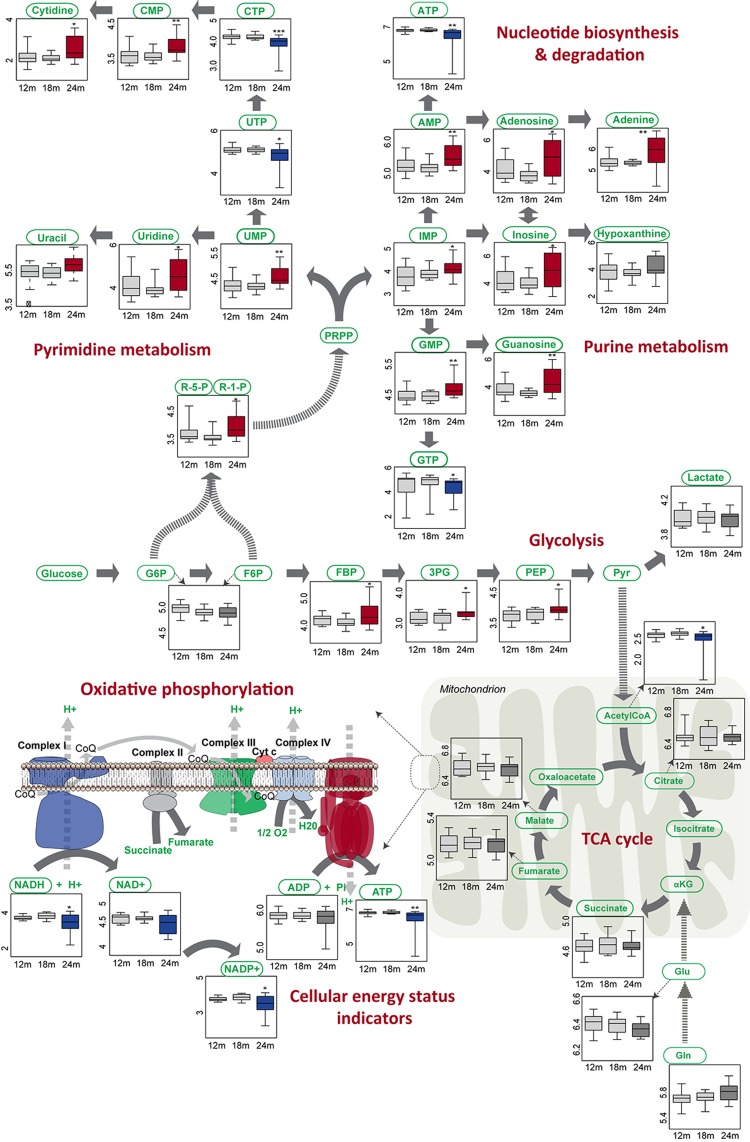
Metabolite level changes in core metabolism of aged mouse brain Significant changes (p ≤ 0.01) at the metabolite levels as validated by targeted quantification (Table S7) are represented by Box-Whisker plots where the blue colored boxes imply significant metabolite depletion and red colored boxes imply significant metabolite increase or accumulation. Additional changes indirectly related to the above presented pathways are shown in Figure S6. Non-significant changes are represented in grey tones. Box and whisker plots display the full range of variation (whiskers: median with minimum _ maximum; boxes: interquartile range). Y-axis is represented as a log_10_ of metabolite area.

Metabolic drift was primarily identified with metabolites involved in central carbon pathways, the changes that were validated by targeted quantification of identified altered metabolites and interconnected pathways. Pathway mapping of the dysregulated metabolites high-lighted altered energy metabolism, including oxidative phosphorylation, glycolysis and nucleotide biosynthesis and degradation. Compromised cellular energy status was marked by NAD decline, increased AMP/ATP ratio and significant imbalance in nucleotide biosynthesis and degradation pathways. This significant drift in nucleotide metabolism was reflected in the accumulation of purines, pyrimidines, nucleosides and nucleoside monophosphates and depletion of nucleoside tri-phosphate levels (Figure 5, Figure S6). Interestingly, the TCA cycle was not dysregulated and mitochondrial dysfunction appears to be related mainly to imbalance in oxidative phosphorylation.

## DISCUSSION

In this model of brain aging, we have found evidence for a compromised central energy metabolic state, as well as changes in nucleotide biosynthesis and degradation. This was reflected in the significant metabolite variations in opposing directions, within the entire metabolite pool and within the specific metabolic pathways. The observed phenomenon can be characterized by loss of normal homeostatic mechanisms, and termed drift-variance. In physiological terms, this drift-variance can be explained as an increasing deviation as homeostasis control is diminished, essentially a failure to maintain steady state levels following a response to external stimuli. These findings are consistent with the theory of metabolic stability of regulatory networks, which claims that the capacity of cells to maintain stable concentrations of critical metabolites (including reactive oxygen species) is a prime determinant of life span [32, 33]. According to this theory and our observations of metabolic drift in the aged brain, the capacity of cells to respond appropriately to stimuli of internal or external origin by successfully moving back to the metabostasis could be a critical factor in the regulation of longevity of brain function [31, 33]. We also note an increased variability in the metabolite levels in the oldest mice, consistent with the increased variability found in a number of physiological parameters including cognitive processes [34].

It has been demonstrated that the imbalance in oxidative phosphorylation (NAD decline, increased AMP/ATP ratio) leads to impaired mitochondrial electron transfer and active respiration thus causing the decrease in mito-chondrial energy-transducing capacity.

Compromised mitochondrial energy metabolism significantly affects neuronal activity and plasticity. Functionally, loss of neuronal plasticity or failure to adapt to decline in energy metabolism is viewed as a hallmark of age-related neurodegenerative diseases and directly associated with the age-dependent cognitive decline. This is one of the main reasons why brain energy metabolism is revisited in many ongoing physiology and behavioral studies. A gradual decline in energy metabolism during aging has been demonstrated by several functional neuroimaging techniques (Lin and Rothman 2014, Yin et al. 2014). PET scanning in aging mice revealed decreases in the cerebral metabolic rate of glucose, and proton (1H) MR spectroscopy (MRS) in the same animals revealed decreases in energy metabolites, correlated with decreases in spatial working and reference memory [35].

The hippocampal proteomic analysis implicated alterations in nucleobase-containing metabolites and revealed potential mechanisms behind such metabolic alterations. The nucleoside diphosphate kinases that play a major role in the synthesis of nucleoside triphosphates other than ATP from nucleoside diphosphates were determined to be significantly downregulated (Nme3, log_2_ = −1.2 and Nme4, log_2_ = −2.6) within the hippocampus of 24- compared to 12-month old mice, consistent with the observed lower levels of the triphosphate forms of these purines and pyrimidines. Further, increased expression of guanylate cyclase 1, soluble, beta 3 (Gucy1b3, log_2_ = 1.9), which catalyzes the conversion of GTP to cGMP would be expected to contribute to the decreased levels of GTP. The proteomics data also revealed decreased expression of the purine recycling enzyme hypoxanthine phosphoribosyltransferase 1 (Hprt1, log_2_ = −0.5) with age within the hippocampus, consistent with altered purine metabolites. Alterations in AMP, ADP and ATP may be related to changes in the ATPase components Atp1b2 (log_2_ = 0.5) and Atp2b1 (log_2_ = −0.4), as well as Adenylate kinase 4 (Ak4, log_2_ = −1.2).

Our proteomics data revealed decreased expression of Sirt3 in the hippocampus of older animals, which is consistent with the metabolomics observation of decreased ATP levels. Studies in Sirt3 knockout animals revealed reduced ATP levels as well as inhibited complex I activity [36]. Further, Sirt3 regulates the activity of acetyl-CoA synthetase 2, an important mitochondrial enzyme involved in generating acetyl-CoA for the TCA cycle [37]. Thus, loss of Sirt3 protein may contribute to the lower acetyl-CoA levels as well. In addition to loss of Sirt3 protein levels, our metabolomics data suggests that the activity of Sirt3 would be reduced since NAD, which was determined to be lower, is required for its activity.

While we examined three different brain regions, there are multiple cell types present within each region (neurons, astrocytes, oligodendrocytes, microglia). The existence of functionally complementary metabolic profiles in two previously studied cell types, neurons and astrocytes, has already been identified in early neurochemical studies in the 1950s and 1960s [38]. Neurons utilize most of the energy in the brain [39, 40] and express high oxidative capacity [41]. In contrast, astrocytes, although capable of oxidative phosphorylation, predominantly process glucose glycol-lytically and shuttle energy substrates, lactate and pyruvate to neurons for use in oxidative phosphorylation [1, 42]. While this shuttle has considerable experimental support there are also experiments indicating that glucose uptake by neurons, not astrocytes, provides the fuel for neuronal oxidative phosphorylation [43, 44]. Thus our findings have parallels to metabolic reprogramming (Figure 5), and can be interpreted either as occurring within the neuron, or in light of the astrocyte shuttle model instead of within a single cell, involving the astrocyte and the neuron pair within brain tissue, with the astrocyte supplying increased glycolytic substrates to the neuron to compensate for disrupted oxidative energy production with age (decreased oxidative phosphorylation) and meet the metabolic requirements of brain. In this model, the balance within individual cell types, however, can become lost, leading to what we term *metabolic drift* or altered relative metabolite stoichiometry.

Steady state isotopic experiments combined with metabolomic profiling of different cell types (shift in population balance between astrocytes *vs*. neurons) and sub-cellular fractions (e.g. mitochondrial fraction and/or synapses) would offer a more complete understanding of the observed drift phenomenon. These global-untargeted isotope-assisted experiments would be especially useful to trace the isotope enrichment across the broad range of metabolites in order to determine the directionality of the observed changes and pathway contribution in the utilization of specific substrates, such as 13C labeled glucose for example. In addition, the recently developed global metabolomic approach of arteriovenous blood analysis [45] could yield direct and real time insight to the aging brain.

## MATERIALS AND METHODS

### Sample collection

Male mice (C57BL/6) were obtained from the National Institute on Aging rodent colonies according to annotation standards for age classification [46]. All protocols were implemented in accordance with NIH guidelines and approved by the Institutional Animal Care and Use Committee at the College of Medicine, University of Nebraska Medical Center. All animals were housed in a controlled room with a constant 12- hour light/dark cycle. Animals were fed NIH31 diet *ad libitum* while housed at the NIA and one month before sacrifice were shipped to UNMC where they were fed Harlan #7912 irradiated rodent chow *ad libitum*. Focused beam microwave irradiation (FBMI) was applied (following brief exposure to 1.5% isoflurane for anesthetic induction) in mice to deliver instantaneous euthanasia and quench the metabolism efficiently prior to brain dissection. As a “quality control” the ^1^HRMS spectra were acquired in the central brain region in order to screen brains for further analysis. The appearance of a large lactate peak was the primary criterion for sample exclusion from further metabolomic and proteomic analysis, as is shown in Figure S1 (for detailed description of FBMI procedure please see Supplemental Information).

### Metabolite and protein extraction

Dissected brain regions (hippocampus, caudoputamen, and frontal cortex) were extracted using a MeOH:H_2_O (4:1, v/v) solvent mixture as a best compromise to precipitate proteins and efficiently extract water-soluble and lipid metabolites. A volume of 500 μL of cold solvent was added to 10 mg of tissue, vortexed for 30 s and incubated in liquid nitrogen for 1 min. The samples were then allowed to thaw at room temperature and sonicated for 5 min. This cycle of tissue disruption was repeated two times for three rounds total. To precipitate proteins the samples were incubated for 1 hour at −20° C, followed by 15 min centrifugation at 17,000 x g at 4° C. The resulting supernatant was removed and evaporated to dryness in a vacuum concentrator. The protein pellets were evaporated to dryness in a vacuum concentrator and lysed in 100 mM Tris-HCl with 4% (w/v) SDS and 0.1 M DTT, pH 7.6 using brief sonication and incubation at 95° C for 5 min. The Pierce 660 nm Protein Assay (Thermo Scientific) was used to determine protein concentration, as a reference for metabolite reconstitution. The dry metabolome extracts were reconstituted in ACN:H_2_O (1:1, v/v) normalized by the sample's protein level, sonicated for 30 sec, and centrifuged 15 min at 13000 rpm and 4° C to remove insoluble debris. The supernatants were transferred to HPLC vials and stored at −80° C prior to LC/MS analysis.

### Proteome analysis

The protein lysates from the 12- and 24-month old mouse hippocampus were analyzed using SWATH-MS Data Independent Acquisition (DIA), following the library generation (for detailed description of spectral library creation please see Supplemental Information). Prior to DIA mass spectrometry analysis, the protein lysates (50 μg) from the mouse hippocampus were digested with trypsin using the FASP method [47]. The resultant peptides were desalted using Oasis MCX cartridges and then quantified using the Scopes method [48]. The unfractionated samples of peptides (2 μg) from the 12- and 24-month old mouse hippocampal lysates for each brain hemisphere (right and left) were analyzed in sextuplet (six biological replicates per age group) using SWATH-MS DIA. LC-MS/MS was conducted using an AB SCIEX TTOF mass spectrometer coupled with an Eksigent NanoLC-Ultra 1D plus and nanoFlex cHiPLC system (Eksigent). Samples were loaded onto a 75 μm x 0.5 mm ChromXP C18-CL 3 μm 120 Å trap column (Eksigent), washed with 98:2 HPLC water with 1% FA for 10 minutes and then eluted through a 75 μm x 15 cm ChromXP C18-CL 3 μm 300 Å analytical column (Eksigent) at 300 nL/min with 98:2 HPLC water with 1% FA using a 90 minute linear gradient of 0-60% ACN with 1% FA. Autocalibration of spectra occurred after acquisition of every 4 samples using dynamic LC-MS and MS/MS acquisitions of 25 fmol β-galactosidase. Experimental samples (hippocampal peptides) were subjected to cyclic DIA of mass spectra using 25 Da swaths in a similar manner to previously established methods [49].

### Proteome data processing and analysis

All of the fragment ion chromatograms were extracted and automatically integrated with PeakView (v.2.1 Beta). The raw peak areas as reported by PeakView were used for all the quantification calculations with no data processing (either de-noising or smoothing) of any kind applied to the extracted ion chromatograms. To calibrate retention times, synthetic peptides (BiognoSYS) were spiked-in the samples in accordance with previously published work [50], we selected 5 peptides and 5 transitions for quantitative analysis and targeted data extraction for each peptide was performed. Briefly, for each peptide the fragment ion chromatograms were extracted using the SWATH isolation window set to a width of 10 min and 50 ppm accuracy for quantification purposes in accordance with previously established protocols [49, 50]. Samples were normalized to the median peak ratios using MarkerView software (v.1.2.1.). The Pearson's correlation coefficient, r, between the right and left hemisphere for each mouse was calculated revealing strong correlation at both ages (average r = 0.93 (12 mo) and 0.97 (24 mo)) demonstrating the ability to quantify proteins from the hippocampus following FBMI with great precision. For subsequent analyses, the protein values for each hemisphere were averaged for each mouse. We also found strong correlation between biological replicates (average r = 0.97 (12 mo) to 0.98 (24 mo)) further supporting our use of SWATH-MS for hippocampal proteome quantification. The data was uploaded to the Cyber-T Web server (http://cybert.ics.uci.edu/) [51], which implements a t-test using a Bayesian regularization method for quantitative mass spectrometry analysis and multiple tests corrections were employed to derive the Posterior Probabilities of Differential Expression (PPDE) and perform Benjamini and Hochberg (BH) corrections. Cutoffs for proteins deemed as significantly differentially expressed were *p* values ≤ 0.05, PPDE values ≥ 0.95, and BH corrected *q* values (FDR) ≤ 0.05. All correlation analysis, linear fits, and frequency distributions were completed using Prism (GraphPad Software). The protein annotation through evolutionary relationship (PANTHER: http://www.pantherdb.org/) classification system (version 9.0) functional classification tool was used to classify lists of proteins according to function via annotation with the Gene Ontology (GO) term biological processes (BP). Ingenuity Pathway Analysis (IPA: Ingenuity Systems; http://www.ingenuity.com/) Metabolic Pathways within the Canonical Pathways tool to determine the likelihood that the association between a set of focus genes in our experiment and a pathway is due to random chance (the smaller the right-tailed Fisher Exact Test p-value, the less likely that the association is random).

### Untargeted metabolome profiling

Samples were analyzed by HILIC ESI-Q-TOF/MS as previously described [52], in MS^1^ mode. Tissue extracts from hippocampus, caudoputamen, and frontal cortex (individual extracts pooled from both hemispheres) from 6 individuals at each time point (3 regions x 6 specimen or a total of 18 samples per time point) were analyzed on 6550 iFunnel QTOF mass spectrometer (Agilent Technologies) interfaced with 1200 HPLC system (Agilent Technologies). No differences were observed between the left and right hemisphere (using mixed linear effect regression model), thus, due to the limited sample amount the final analysis (untargeted and targeted) as reported in results were performed on individual extracts pooled from both hemispheres. Samples were analyzed using a Luna Aminopropyl, 3 μm, 150 mm × 1.0 mm I.D. HILIC column (Phenomenex). The mobile phase was composed of A = 20 mM ammonium acetate and 20 mM ammonium hydroxide in 95% water and B = 95% acetonitrile [53]. The linear gradient elution from 100% B (0−5 min) to 100% A (45−50 min) was applied (A = 95% H2O, B = 95% ACN, with appropriate additives). A 10 min postrun was applied for HILIC, to ensure the column re-equilibration and maintain the reproducibility. The flow rate was 50 μL/min, and the sample injection volume was 2 μL. ESI source conditions were set as follows: dry gas temperature 200°C and flow 11 L/min, fragmentor 380 V, sheath gas temperature 300°C and flow 9 L/min, nozzle voltage 500 V, and capillary voltage −2500 V in ESI negative mode. The instrument was set to acquire over the m/z range 50−1000, with the MS acquisition rate of 2 spectra/s. For the Auto MS/MS the isolation width was set as narrow (∼ 1.3 m/z), with a MS acquisition rate of 350ms and MS/MS acquisition rate of 75ms. In each cycle (1.2s), 10 precursor ions were chosen for fragmentation at collision energy (CE) of 20 V and 40V. Selected precursor was excluded after the MS/MS data was acquired 3 times and released after 0.15 minutes. Additional Auto LC/MS/MS analyses were performed using an HPLC system (1200 series, Agilent Technologies) coupled to TTOF 5600 (Q-TOF, AB Sciex), in DDA mode. In each cycle, 15 precursor ions were chosen for fragmentation at collision energy (CE) of 30 V (15 MS/MS events with product ion accumulation time of 50 msec each) [54] ESI source conditions were set as following: Ion source gas 1 as 15, Ion source gas 2 as 10, Curtain gas as 10, source temperature 550°C, Ion Spray Voltage Floating (ISVF) 5500V or −4500V in positive or negative modes, respectively. LC conditions (column, mobile phase and gradient) were the same as described for HILIC untargeted profiling.

### Targeted multiple pathway analysis

Quantitation of metabolites from multiple central core pathways was performed by HILIC ESI-QqQ/MS in Dynamic multiple reaction monitoring mode (MRM) as described in more details in Supplemental Information.

Metabolome data processing and analysis. Raw LC/MS data were converted to mzXML files using ProteoWizard MS Convert version 3.0.7529 [55]. mzXML files were uploaded to XCMS Online web platform for data processing (http://xcmsonline.scripps.edu) including peak detection, retention time correction, profile alignment, and isotope annotation [56, 57]. Data were processed as a multi-group experiment and the parameter settings were as follows: centWave for feature detection (Δ m/z = 15 ppm, minimum peak width = 10 sec and maximum peak width = 120 sec); obiwarp settings for retention time correction (profStep = 1); and parameters for chromatogram alignment, including mzwid = 0.015, minfrac = 0.5 and bw = 5. The relative quantification of metabolite features was based on EIC (Extracted Ion Chromatogram) areas. One-way Analysis of Variance (ANOVA) and post-HOC Tukey test was used to filter out the significantly altered metabolite features over time (12 months - mature adult, 18 months - old and 24 months - aged). The results output, including metabolite feature table, extracted ion chromatograms and multi-group cloud plot, were exported directly from XCMS Online. One extract of hippocampus at 24 months was excluded from the analysis as an outlier. Mummichog computational algorithm based on BioCyc pathway database (http://biocyc.org) [58] was used to accelerate metabolite feature filtering, characterize the biological activity network and predict metabolite identifications [59]. After filtering out the isotopes, adducts, multiple charged species and in-source fragments, only the corresponding deprotonated monoisotopic features were used in further analysis. Metabolite identification was validated using the accurate mass (within 5 ppm), retention time and MS/MS data. Accurate masses were searched against databases METLIN and HMDB. The identifications were made by matching the acquired MS/MS data for altered metabolites in brain tissue extract against MS/MS data recorded for standards, assembled in in-house developed and online available METLIN database (https://metlin.scripps.edu/index.php) [60, 61]. MS/MS matches are provided in the Supplemental Information (Figure S5). Mixed linear effect regression model was used to further test different factors (time, brain hemisphere, brain region) for the variation of identified altered metabolites in aged mice. Altered metabolome variation pattern was further explored using heat map combined with Hierarchical Cluster Analysis in R version 3.1.2 (gplots library). A z-transformation was performed on all peak areas to scale the data. Hierarchical clustering analysis (HCA) of metabolite patterns was performed using Euclidean distance matrix and the complete linkage method. Pathway enrichment analysis were performed with MetaboAnalyst a web server designed for comprehensive metabolomics data analysis [62].

### Metabolic drift calculation

We had 6 metabolite abundance values for each metabolite (6 biological replicates per brain regions per time point) for each age: 12 months, 18 months and 24 months. A mean metabolite abundance was calculated for each individual metabolite for each age group. The metabolic drift was calculated as a log of metabolite fold change from old (18 and 24 months) to reference adult stage (12 months). To calculate the drift-variance value within the adult reference stage group (12 months) one biological replicate in that group was used as reference, the others as experimental samples.

## SUPPLEMENTAL INFORMATION FIGURES AND TABLE


