# Supplementary material for: Metabolic drift in the aging brain
Source: Aging (Albany NY). 2016 May 15;8(5):1000–13. doi: 10.18632/aging.100961 (PMC4931850; doi:10.18632/aging.100961)
Supplement: Supplementary file 1 [file aging-08-1000-s001.pdf]

SUPPLEMENTAL INFORMATION

Supplemental Methods

FBMI euthanasia and ^{1}H MRS. Mice were anesthetized by inhalation of 1-2% isoflurane in oxygen and aligned in the water-jacketed animal holder for microwave irradiation in a Muromachi Microwave Fixation System (10 kW model). The irradiation times varied from 800 to 820 ms at constant 4.9 kW depending on the body weight (g) of each mouse, as determined previously [1]. The brain was then isolated and split into hemispheres. Three regions (hippocampus, caudoputamen, midbrain and frontal cortex) were dissected following anatomical boundaries and immediately flash frozen in liquid nitrogen prior to storage at -80° C. Proton magnetic resonance spectroscopy (^{1}H MRS) was performed on post-mortem, thawed midbrain tissues to validate the degree of metabolite stability by comparing post-mortem metabolite levels to those characterized in live animals done in other studies [1, 2]. Nine millimeter cubic volume, single voxel localized spectra were acquired on brain tissue placed in perflouropolyether oil (Fomblin, Fisher Scientific, Pittsburg, PA) to measure metabolite signals using point resolved spectroscopy (PRESS) sequence. Spectra were acquired with a repetition time of 4 seconds, echo time of 50 ms, 64 averages, using a custom built solenoid coil on a 7 T/16 cm Bruker Pharmascan MRI/MRS system. Brains with increased lactate signals were eliminated from further analysis (Figure S1). Six specimens at each time point (12, 18 and 24 months) were selected for further brain proteome and metabolome analysis.

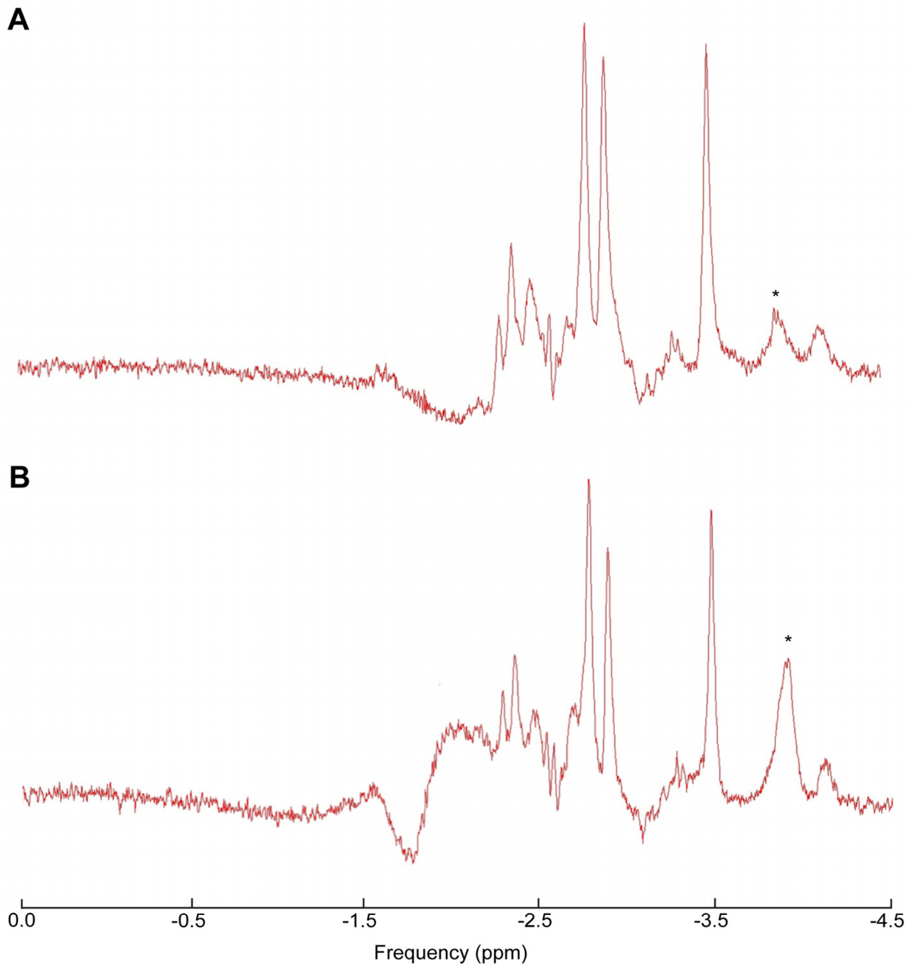

Figure S1. Representative single voxel ^{1}H MRS spectra of the mouse hippocampus post-microwave fixation used to validate sample selection for metabolomics. The ^{1}H MRS spectra demonstrate the effect of adequate (**A**) and inadequate (**B**) heat stabilization on lactate (*).

.

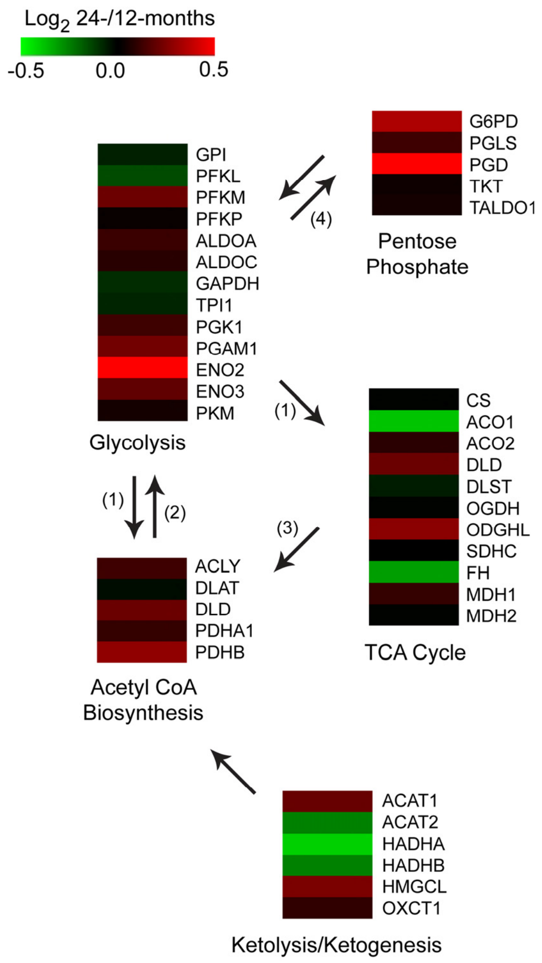

Figure S2. Heatmaps showing the protein expression changes from 12- to 24-months in the mouse hippocampus across the metabolic pathways that comprise the generation of precursor metabolites and energy. Legend: 1 – pyruvate, 2 – oxaloacetate, 3 – citrate, 4 – D-glucose-6P / Glyceraldehyde-3P, 5 – NADH/$FADH_2$ Related to Figure **2.**

.

In regards to the anesthesia, mice were placed into an induction chamber with 1.5% isoflurane in 100% oxygen for 10 minutes prior to being placed into the holder for subsequent focused-beam microwave irradiation (FBMI, to provide instantaneous euthanasia and metabolism quenching due to the heat inactivation of enzymes). Within the FBMI holder, mice were free breathing room air. While we did not measure arterial PO2, we have had extension experience with isoflurane during rodent MRI. In our experience, breathing rate is typically high after induction (50-90 breaths per minute) and does not slow down until about 15 minutes into the scanning session during MRI experiments with continued isoflurane exposure. In addition, parallel studies of physiology have shown that, with extended periods of anesthesia, keeping the breathing rate at 50 pbm or greater, there is no deoxygenation of the arterial blood as measured by pulse oximetry and there is a normal physiological response to 5% CO2 including increased breathing rate and increased cerebral perfusion during scanning periods exceeding three hours (our unpublished data). While we have not found studies comparing young and old mice, one study did indeed find a decrease in pO2, and increase in pCO2, after 2-3 hours of isoflurane anesthesia, far longer than the short induction performed here [3]. Thus increased anaerobic metabolism is an unlikely consequence of a short period of isoflurane.

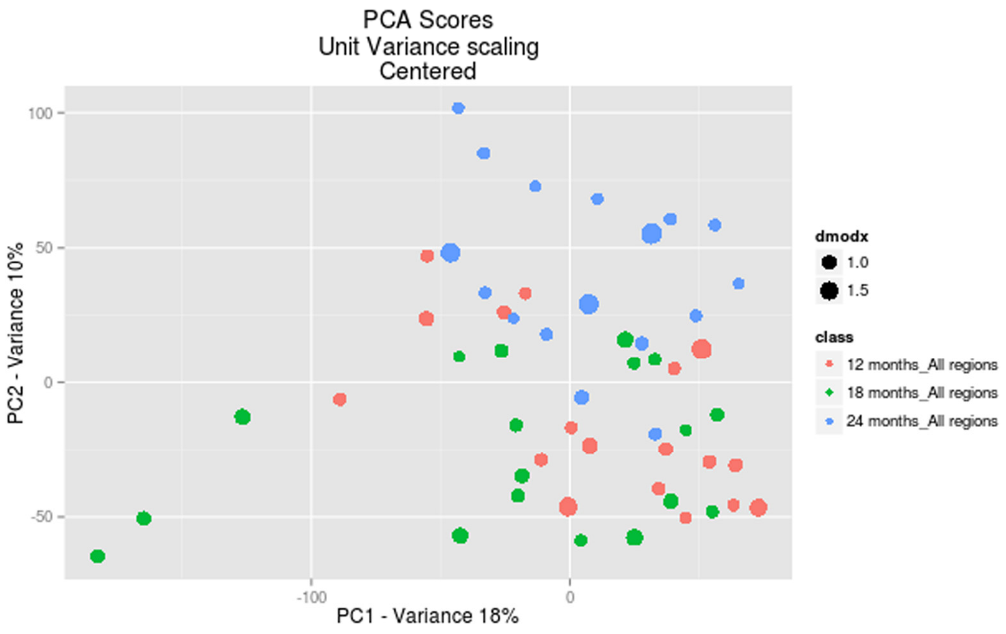

Figure S3. Principal component analysis of brain metabolome extracts across three different time points. Scoring plot demonstrates the separation of aged mice group as a function of differences in metabolic profiles.

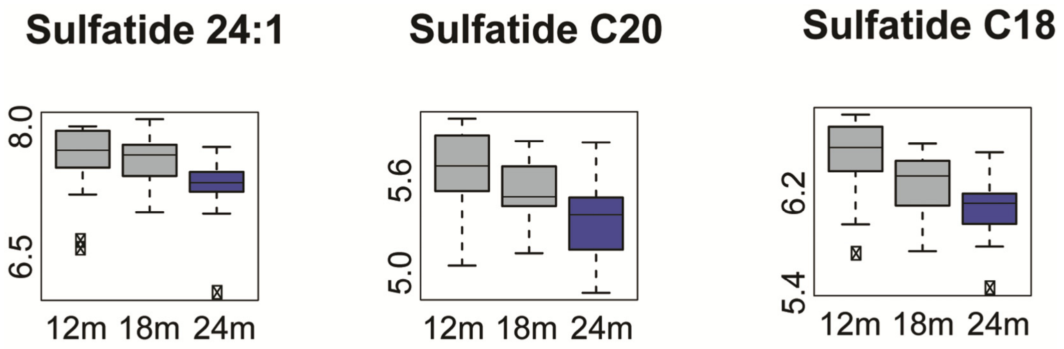

Figure S4. Sulfatide level changes in the aged mouse brain. Significant changes in the levels of specific brain lipids sulfatides ($p \leq 0.01$) are represented by Box-Whisker plots where the blue colored boxes imply significant metabolite depletion. Box and whisker plots display the full range of variation (whiskers:median with minimum _ maximum and outliers; boxes: interquartile range). Y-axis is represented as a $\log_{10}$ of metabolite area

Proteome reference library generation. The hippocampal protein fractions from both hemispheres of mice at each age were used to generate the SWATH-MS reference spectral library that was used to extract quantitative levels of proteins from the hippocampus of 12- and 24-month old mice. Protein lysates were prepared from each hemisphere from two mice, 12- and 24-months old, and mixed in equal amounts. This lysate mixture was ali quoted into 100 µg samples for trypsin digestion using the filter-aided sample preparation (FASP) method [4]. The peptides were desalted using Oasis mixed-mode weak cation-exchange (MCX) cartridges following the manufacturer's protocols. The resulting peptides were quantified by absorbance at 205 nm [5]. Peptides (35 µg) were fractionated into 12 fractions from pH 3 to 10 (low-resolution kit) by isoelectric focusing using an Agilent 3100 OFFGEL Fractionator (Agilent Technologies).

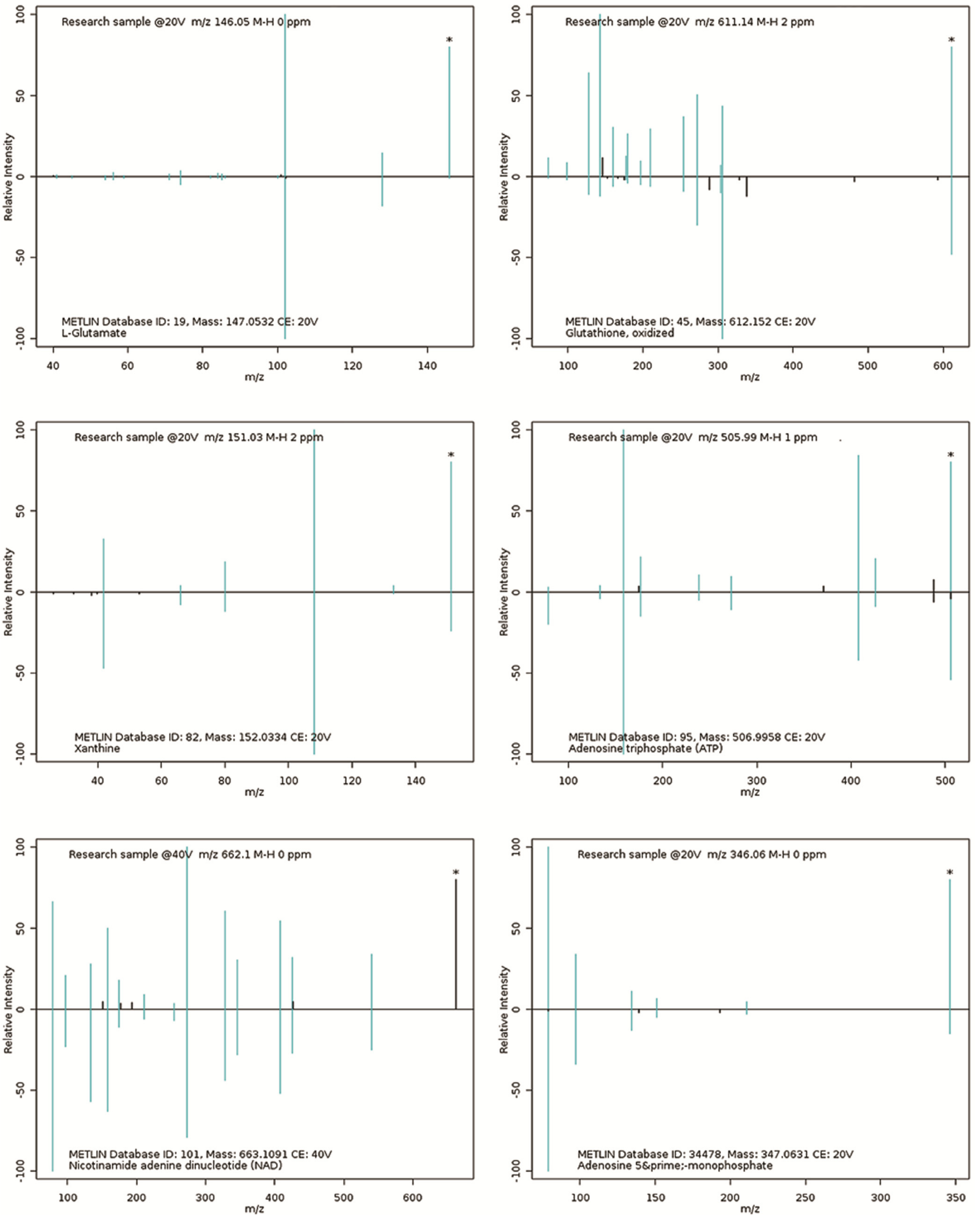

Figure S5. Matching of tandem mass spectrometry data acquired by autonomous IDA (Information Dependent Analysis for sequential MS1 and MS2 acquisition "on fly") against the standard METLIN metabolite database, to validate the metabolite identification in the global-untargeted data analysis.

Fractionated peptides were cleaned and prepared for mass spectrometry using Pierce C-18 PepClean Spin Columns (Thermo Fisher). Samples were dehydrated with a Savant ISS 110 SpeedVac Concentrator (Thermo Fisher) and resuspended in 6 µL of 0.1% FA for LC-MS/MS analysis. In order to generate the SWATH-MS reference spectral library the prepared fractions were subjected to traditional Data-Dependent Acquisition (DDA) as described previously [6]. Briefly, one precursor scan followed by fragmentation of the 50 most abundant peaks was performed. Precursor peaks with a minimum signal count of 100 were dynamically excluded after two selections for 6 seconds within a range ± 25 mDa. Charge states other than 2-5 were rejected. Rolling collision energy was used. All DDA LC-MS/MS files were searched in unison using ProteinPilot as described above [6]. Combined results yielded a library of 456,807 spectra representing 41,515 peptides and 4,671 proteins identified with high confidence (greater than 99%) that passed the global FDR from fit analysis using a critical FDR of 1%.

Targeted validation. Quantitation of metabolites of interest was performed using an HPLC system (1290 Infinity, Agilent Technologies) coupled to ion-Funnel Triple quadrupole 6490 (QqQ, Agilent) mass spectometer. It was operated in Dynamic multiple reaction monitoring mode (MRM), where the collision energies and product ions (MS2 or quantifier and qualifier ion transitions) were pre-optimized for each metabolite of interest (Table S7). Cycle time was 500 ms, and the total number of MRM's was 137. ESI source conditions were set as following: gas temperature 225 °C, gas flow 15 L/min, nebulizer 35 psi, sheath gas 400 °C, sheath gas flow 12 L/min, capillary voltage 2500V and nozzle voltage 0V in ESI negative mode. The analyses were performed on the same type of Phenomenex aminopropyl column as for untargeted analysis, but the larger size 150mm x 2mm, with the same mobile phases, at the 350 µL/min flow rate. Metabolites were targeted in a negative ionization mode, using the gradient from 95 % B (0-2 min) to 10% B (15 min) to 0% B (17-20 min). A 4 min column re-equilibration was applied at the initial solvent composition, to ensure the reproducibility. The injection volume was 2 µL for all analyzed tissue extracts. Standard compound mixtures were used for method optimization, calibration and as a quality control. The ion response for each standard solution was determined by integrating the area of the quantifier transitions listed in Table S7 for each compound (Agilent QQQ Quantitative Analysis).

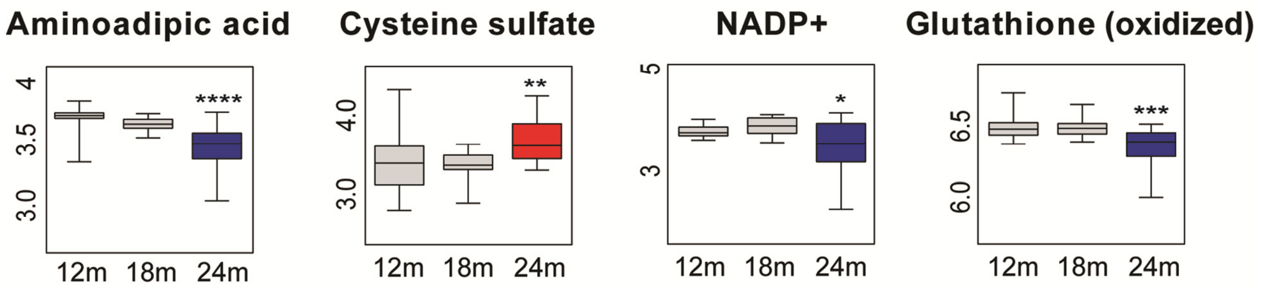

Figure S6. Additional changes in metabolite levels indirectly related to the changes in core pathways as presented in Figure 4. Box and whisker plots display the full range of variation (whiskers: median with minimum _ maximum; boxes: interquartile range). Y-axis is represented as a log_{10} of metabolite area.

Table S7. List of quantified metabolites with matching precursor ions, optimized transition states (quantifier and qualifier ions), and collision energies in negative ionization mode. Related to Figure 5

	Metabolite	Precursor ion (MS1)	Quantifier ion transition (MS2)	Qualifier ion transition(s) (MS2)	Collision energy (V)
1	Acetyl-CoA	808.1	78.9	408	75/38
2	Acetyl-glutamic acid	188.1	128	102.1	10/14
3	Adenine	134	107	92	18/22
4	Adenosine	266.1	134.1	107	26/40
5	ADP	426	134.1	158.9/78.9	22/30/74
6	alpha-ketoglutarate	145	101	57	6/10
7	Aminoadipic acid	160.1	116.1	142.1	14/10

8	AMP	346.1	78.9	96.9/134.1	46/26/42
9	Arginine	173.1	131.1	156.1	10/10
10	ATP	505.9	158.9	408/78.9	26/22/75
11	CDP	402	158.9	384/78.9	22/18/66
12	Citric acid	191	111	87	6/18
12	CMP	322	78.9	96.9	34/30
13	CTP	481.9	158.9	384/78.9	38/22/66
14	Cysteine sulfate	199.9	136	80.9/74	10/10/14
15	Cytidine	242.1	109	42	10/26
16	Fructose-1,6-bisphosphate	338.9	96.9	241/78.9	14/14/74
17	Fructose-6-phosphate	259	78.9	138.9	54/14
18	Fumarate	115	71	27	2/10
19	GDP	442	150	158.9/78.9	26/30/75
20	GDP-mannose	604.1	424	158.9/78.9	26/34/46
21	Glutamate	146	128	102.1	6/10
22	Glutamine	145.1	109	127.1	10/6
23	Glyceraldehyde-3P	168.9	150.9	96.9/78.9	6/2/18
24	Glycerate-3P	184.9	78.9	167.1/96.9	10/10/10
25	GMP	362.1	78.9	211/133	26/14/46
26	GTP	521.9	424	158.9/ 78.9	22/26/75
27	Guanosine	282.1	150	133	18/30
28	Hypoxanthine	135	92	65	14/30
29	IMP	347	96.9	135/ 78.9	22/38/74
30	Inosine	267.1	135	108	26/46
31	Lactate	89	43	41	10/20
32	Malate	133	115	71	6/14
33	Methionine	148	47	32	14/66
34	NAD+	662.1	540.1	158.9/ 78.9	6/50/75
35	NADH	664.1	397	408/ 78.9	30/38/75
36	NADP+	742.1	620	408	10/35
37	Oxidized glutathione	611.1	306.1	272.1/143	22/30/46
38.	Phosphoenol pyruvate	166.9	78.9	62.9	14/75
39	PRPP	388.9	176.9	290.9/78.9	14/10/62
40	Ribose-1-phosphate	229	78.9	138.9/96.9	54/10/10
41	Saccharopine	275.1	257.1	196.1/145.1	10/18/26
42	Succinate	117	73	99	10/22
43	Sulfoacetic acid	138.9	94.9	79.9	10/26
44	Tryptophan	203.1	116.1	142.1	10/14
45	Tyrosine	180.1	163	119.1	10/14
46	UDP	402.9	158.9	111/78.9	26/18/75
47	UDP-galactose	565.1	323	158.9/78.9	22/54/75
48	UDP-N-acetyl-glucosamine	606.1	78.9	272.9/158.9	70/34/62
49	UMP	323	96.9	111/78.9	22/30/58
51	Uric acid	167	124	96/41.9	14/18/50
52	Uridine	243.1	200	110	6/10
53	UTP	482.9	158.9	384.1/78.9	34/18/74
54	Xanthine	151	108	41.9	18/30

Supplemental References

1. Epstein AA, Narayanasamy P, Dash PK, High R, Bathena SP, Gorantla S, Poluektova LY, Alnouti Y, Gendelman HE and Boska MD. Combinatorial assessments of brain tissue metabolomics and histopathology in rodent models of human immunodeficiency virus infection. J Neuroimmune Pharmacol. 2013; 8:1224-1238.

2. Ivanisevic J, Epstein AA, Kurczy ME, Benton PH, Uritboonthai W, Fox HS, Boska MD, Gendelman HE and Siuzdak G. Brain region mapping using global metabolomics. Chem Biol. 2014; 21:1575-1584.

3. Stratmann G, Sall JW, Bell JS, Alvi RS, May L, Ku B, Dowlatshahi M, Dai R, Bickler PE, Russell I, Lee MT, Hrubos MW and Chiu C. Isoflurane does not affect brain cell death, hippocampal neurogenesis, or long-term neurocognitive outcome in aged rats. Anesthesiology. 2010; 112:305-315.

4. Wisniewski JR, Zougman A, Nagaraj N and Mann M. Universal sample preparation method for proteome analysis. Nat Meth. 2009; 6:359-362.

5. Scopes RK. Measurement of protein by spectrophotometry at 205 nm. Anal Biochem. 1974; 59:277-282.

6. Villeneuve LM, Stauch KL and Fox HS. Data for mitochondrial proteomic alterations in the developing rat brain. Data in Brief. 2014; 1:42-45.
